# Dupilumab treatment outcomes in bullous pemphigoid: a systematic review and single-arm meta-analysis

**DOI:** 10.3389/fimmu.2026.1651543

**Published:** 2026-02-06

**Authors:** Yudi Chen, Kailv Sun, Jianmin Chang

**Affiliations:** Department of Dermatology, Beijing Hospital, National Center of Gerontology, Institute of Geriatric Medicine, Chinese Academy of Medical Sciences, Beijing, China

**Keywords:** biologics, bullous pemphigoid, dupilumab, single-arm meta-analysis, systematic review

## Abstract

**Background:**

Bullous pemphigoid (BP) is the most common autoimmune subepidermal bullous disease of the skin. Novel biologic agents represent a potential therapeutic option. We explore the use of dupilumab in the treatment of BP.

**Methods:**

Relevant studies published up to Oct. 20th, 2025 were systematically searched using PubMed, Web of Science, Embase, and Cochrane Library. Proportion rates of complete response and disease control were analyzed to determine treatment effects. Data were quantitatively synthesized using a random-effects meta-analysis. Meanwhile, we also conducted statistics on adverse events.

**Results:**

A total of 587 patients from 24 studies were included. Pooled analysis revealed a complete response rate of 68% (95% CI 60%∼78%) and disease control rate of 95% (95%CI 92%~98%) in BP treated with dupilumab with/without other systemic therapy. Notably, complete response rate achieved 63% (95% CI 49%∼81%) in patients with dupilumab without other systemic therapy. A total of 112 adverse events were reported in 97 patients. Most adverse events were mild and did not lead to treatment discontinuation.

**Conclusion:**

This meta-analysis highlights the efficacy and safety of dupilumab in patients with BP, offering valuable evidence to guide future clinical practice.

**Systematic review registration:**

https://www.crd.york.ac.uk/PROSPERO/, identifier CRD420251048550.

## 1 Introduction

Bullous pemphigoid (BP) is the most common autoimmune subepidermal blistering dermatosis, which typically develops in patients older than 70 years (1). The global incidence of BP was reported as 0.0419 per 1000 person-years (2). The severity of itch and cutaneous lesions significantly disturbs the quality of life in affected patients. BP is mediated by tissue-bound and circulating autoantibodies directed against BP antigen 180 (BP180) and BP antigen 230 (BP230) which are hemidesmosomes proteins (1). Recent evidence has linked the pathogenesis of BP to Th2 inflammation, and this relationship is mediated by Th2-related cytokines, such as interleukin(IL)-4 and IL-13 which are found elevated in the skin, sera and blister fluid of BP patients. Studies identified that these Th2-related cytokines could induce IgE production in B cells contributing to the loss of tolerance against BP antigens and eosinophilia (3).

Dupilumab inhibits both IL-4 and IL-13 signaling by blocking the IL-4 receptor alpha subunit. Dual blockade of IL-4 and IL-13 elicits two key anti-inflammatory effects: it not only impedes the migration and subsequent recruitment of eosinophils to inflammatory foci, but also restricts B-cell proliferation and class-switching, ultimately resulting in decreased IgE production (3). Decreased eosinophil infiltration at inflammatory sites markedly mitigates pruritus driven by eosinophil degranulation. Concurrently, diminished IgE synthesis not only abrogates its T-cell activation-promoting capacity, but also attenuates degranulation of eosinophils and mast cells, leading to amelioration of erythema, blisters and pruritus in patients (4).

Systemic corticosteroids are widely used in the treatment of bullous pemphigoid, which is recommended by several clinical practice guidelines (1, 5, 6). However, long-term application of corticosteroids may cause serious side effects, especially in elderly population. New therapeutic pharmacologic biologic agents such as dupilumab can selectively inhibit inflammatory cascade and it may be a safer and effective treatment option. Satisfactory therapeutic effect of dupilumab in BP has been observed in several studies (please refer to the **Supplementary Table S2**).

To date, there remains a paucity of meta-analyses addressing dupilumab use in BP. No randomized controlled trial (RCT) on dupilumab for BP has been published up till now. Additionally, the potential severity of the disease poses significant challenges for conducting randomized placebo-controlled trials in this patient population. Based on the clinical studies that have been published so far, we conducted a single-arm meta-analysis assessing the efficacy and safety of dupilumab in BP to provide comprehensive evidence for clinical practice.

## 2 Methods

The systematic review was performed following the Preferred Reporting Items for Systematic Reviews and Meta-Analyses (PRISMA) guidelines (7). The study protocol has been registered with PROSPERO (CRD420251048550).

### 2.1 Data sources and search strategy

Two authors (Y.D.C. and K.L.S.) conducted a bibliographic search of all articles published up to Oct. 20th, 2025 using PubMed, Web of Science, Embase, and Cochrane Library. Search terms included combinations of terms related to dupilumab, bullous pemphigoid (BP), and clinical trials. To ensure thoroughness, we also reviewed references from relevant published studies and review articles to address any gaps in the keyword search. The complete search strategy can be found in **Supplementary Table S1**.

### 2.2 Eligibility criteria and study selection

Inclusion criteria were (1): RCTs, single-arm trials, observational studies and case series involving patients with BP regardless of disease severity (2): studies involving dupilumab treatment for BP (3): studies reporting resolution outcomes on biologic treatment. Irrelevant studies were excluded based on the following criteria (1): duplicate studies from the same trials (2): reviews, case reports or case series with sample size less than 5, clinical guidelines, protocols and conference abstracts (3): publications did not report resolution outcome (4): publications written in languages other than English.

Two authors (Y.D.C. and K.L.S.) independently screened all potentially eligible studies. Full texts were assessed for eligibility when abstracts provided insufficient information. Any discrepancies were resolved through group discussion with the senior author (J.M.C.).

### 2.3 Data extraction

For each selected study, the following information was extracted: first author, publication year, study design, region, sample size, sex, age, BP duration, potential trigger, treatment regimens, resolution outcomes (complete remission, disease control, no remission, deterioration), baseline Bullous Pemphigoid Disease Activity Index (BPDAI), and adverse events. If a study didn’t report data on a specific demographic characteristic, the data from that study shall be excluded from the descriptive statistics of this demographic characteristic. And it will be marked as “NR” as shown in **Table 1**, **Supplementary Table S2**.

**Table 1:** Summary of demographic information.

Demographics	N=587
Age, y	
Mean	75.1
Range	35∼101
NR, n (%)	36 (6.1)
Sex, n (%)	
Female	241 (41.1)
Male	310 (52.8)
NR	36 (6.1)
BP duration, months	
Mean	19.2
Range	0.5∼180
NR, n (%)	72 (12.3)
BPDAI at baseline	
Mean	50.6
NR, n (%)	376 (64.1)
Treatment outcomes, n (%)	
Complete response^a^	330 (56.8)
Disease control^b^	439 (90.7)
Drug-induced BP, n (%)	117 (19.9)
immunotherapy-associated	35 (29.9)
DPP-4 inhibitor-associated	32 (27.4)
Diuretic-associated	28 (23.9)
ACEI-associated	18 (15.4)
NR	4 (3.4)
Patients with no other systemic therapy, n (%)	179 (30.5)
Patients with concomitant systemic therapy, n (%)	408 (69.5)
Systemic corticosteroids, n (%)^c^	304 (74.5)
Antibiotics	
Doxycycline, n (%)	20 (4.9)
Minocycline, n (%)	138 (33.8)
Dapsone, n (%)	11 (2.7)
Immunosuppressants	
Methotrexate, n (%)	15 (3.7)
Azathioprine, n (%)	15 (3.7)
Cyclosporine, n (%)	3 (0.7)
Cyclophosphamide, n (%)	1 (0.2)
Mycophenolate mofetil, n (%)	7 (1.7)
Tacrolimus, n (%)	3 (0.7)
Biologics	
Omalizumab	1 (0.2)
Others	
Tripterysium glycosides tablets, n (%)	16 (3.9)
Niacinamide, n (%)	7 (1.7)
Thalidomide, n (%)	1 (0.2)
Albumin, n (%)	2 (0.4)
Immunoglobulin, n (%)	4 (1.0)
Antihistamine, n (%)	1 (0.2)
NR, n (%)	42 (10.3)

a The study of *Jinghui Li 2024* did not report complete response rate (n=6).

b The study of *Planella-Fontanillas 2024* (n=103) did not report disease control rate.

c Proportion of number of patients using each concomitant systemic therapy in all patients with concomitant systemic therapy.

ACEI, angiotensin-converting enzyme inhibitor; BP, bullous pemphigoid; BPDAI, Bullous Pemphigoid Disease Activity Index; DPP-4, dipeptidyl peptidase-4; NR, not reported.

Resolution outcomes on treatment are defined as follows (1). complete remission: The total resolution of BP lesions. The publications use the terms “complete remission”, “complete response”, “complete control”, or “symptom-free” (2). partial remission: Improvement yet lack the complete resolution of BP lesions. The publications use the terms “partial remission”, “partial response”, “improved”, and “clinical improvement” (3). disease control: New lesions cease to form and established lesions begin to heal. Both “complete remission” and “partial remission” can be regarded as “disease control” (4). no remission: No changes in BP lesions. The publications use the terms “no resolution” or “no response” (5). deterioration: Exacerbation of BP lesions.

### 2.4 Risk of bias and statistical analysis

ROBINS-I tool was used to assess risk of bias in non-randomized studies (8). Statistical information, including the single-arm proportion rate of the outcomes and 95% confidence intervals (CIs), was analyzed to determine treatment effects. Heterogeneity was assessed using I² values. Data of resolution outcomes were quantitatively synthesized using a random-effects meta-analysis. All tests were two-sided, and a p-value < 0.05 was considered significant. Sensitivity analyses were conducted to assess the robustness and reliability of the combined results. Analyses were performed using R statistical software version 4.2.3.

## 3 Results

### 3.1 Study characteristics

**Figure 1:**
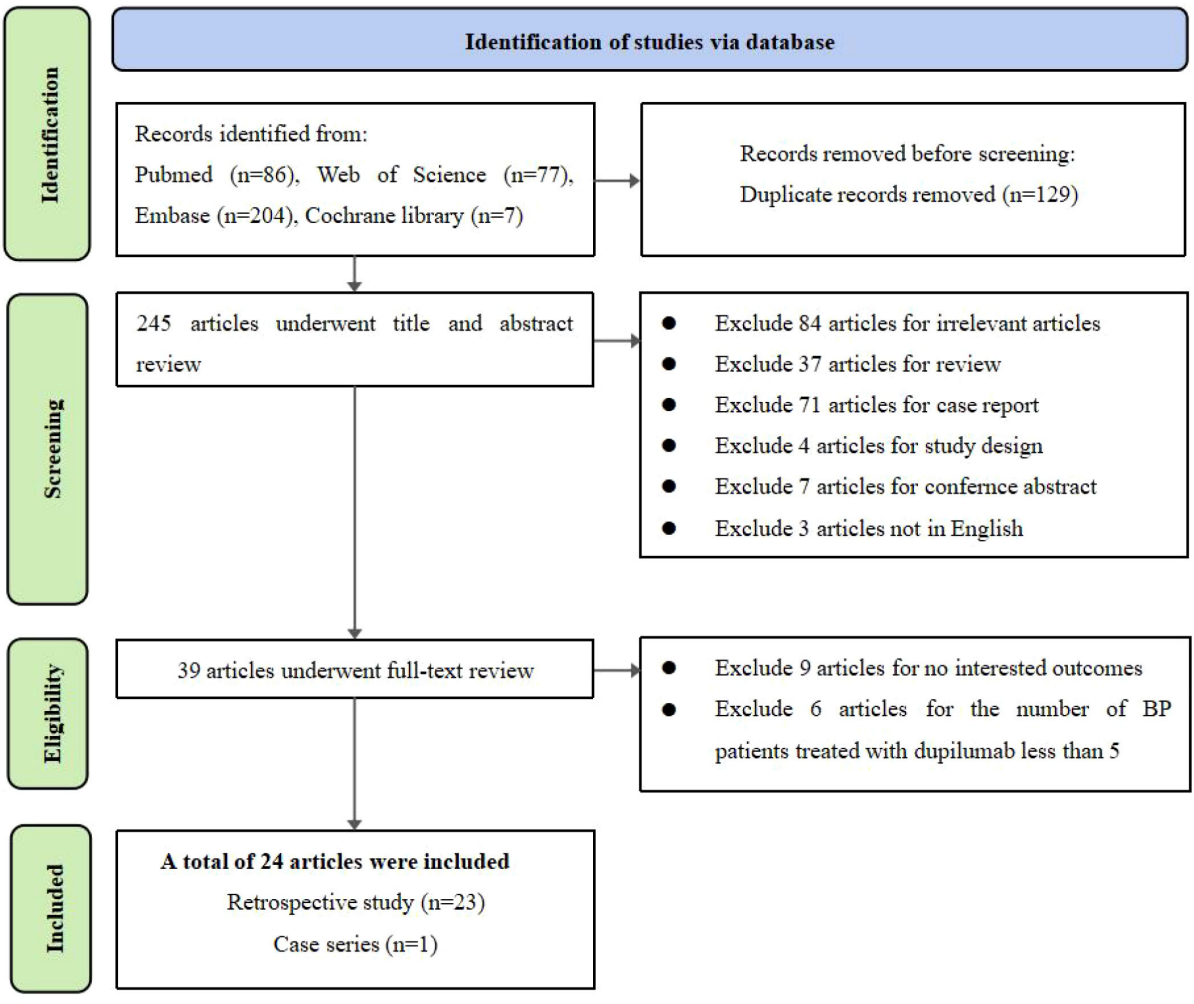
Study flow diagram.

Our search identified 245 potentially relevant non-duplicate articles. All studies were reviewed by title and abstract. Subsequently, 206 articles were excluded because they did not fulfill inclusion criteria. The remaining 39 studies were evaluated in full length. As 9 articles *(Greenberg 2025, Jin 2025, Liang 2025, Huang 2023, Bin 2023, Lukac 2023,Yang 2022, Bur 2022, Phillips 2019)* did not report the proportion or number of BP patients achieved “complete remission” or “partial remission” or “disease control” under dupilumab therapy, these 9 articles were excluded (9–17). Meanwhile, as 5 articles *(Svara 2025, Young 2024, Oren-Shabtai 2023, Merli 2023, Liu 2021)* only contained 1 participate and 1 article *(Lo 2022)* only contained 4 participates receiving dupilumab therapy, these articles were also excluded (18–23). Finally, 24 studies fulfilled all of the eligibility criteria and were included in our systematic review (**Figure 1**). All of the included studies were non-randomized studies, including 23 retrospective studies and 1 case series. Detailed information is summarized in **Supplementary Table S2**. ROBINS-I was utilized for assessing risk of bias of included studies. Detailed information can be found in **Supplementary Table S3**.

### 3.2 Demographic characteristics

The 24 included studies were performed on 587 adult patients with BP. Among these 587 patients (mean age were 75.1 years old, ranging from 35 to 101 years old), 41.1% (n=241/587) were women, 52.8% (n=310/587) were men, and 6.1% (n=36/587) had no gender information. Mean BP duration was 19.2 months (ranging from 0.5 to 180 months). Drug-induced BP were reported in 117 (19.9%) patients, resulting from immunotherapy, dipeptidyl peptidase-4 (DPP-4) inhibitors, diuretics, angiotensin-converting enzyme inhibitors, etc. During dupilumab treatment period, a total of 179 (30.5%) patients received no other systemic therapy. The rest 408 (69.5%) patients received at least one concomitant systemic therapy, including systemic corticosteroids, antibiotics (doxycycline, minocycline, or dapsone), immunosuppressants, tripterygium glycosides tablets, etc. The demographic characteristics are summarized in **Table 1**.

### 3.3 Treatment outcomes

**Figure 2:**
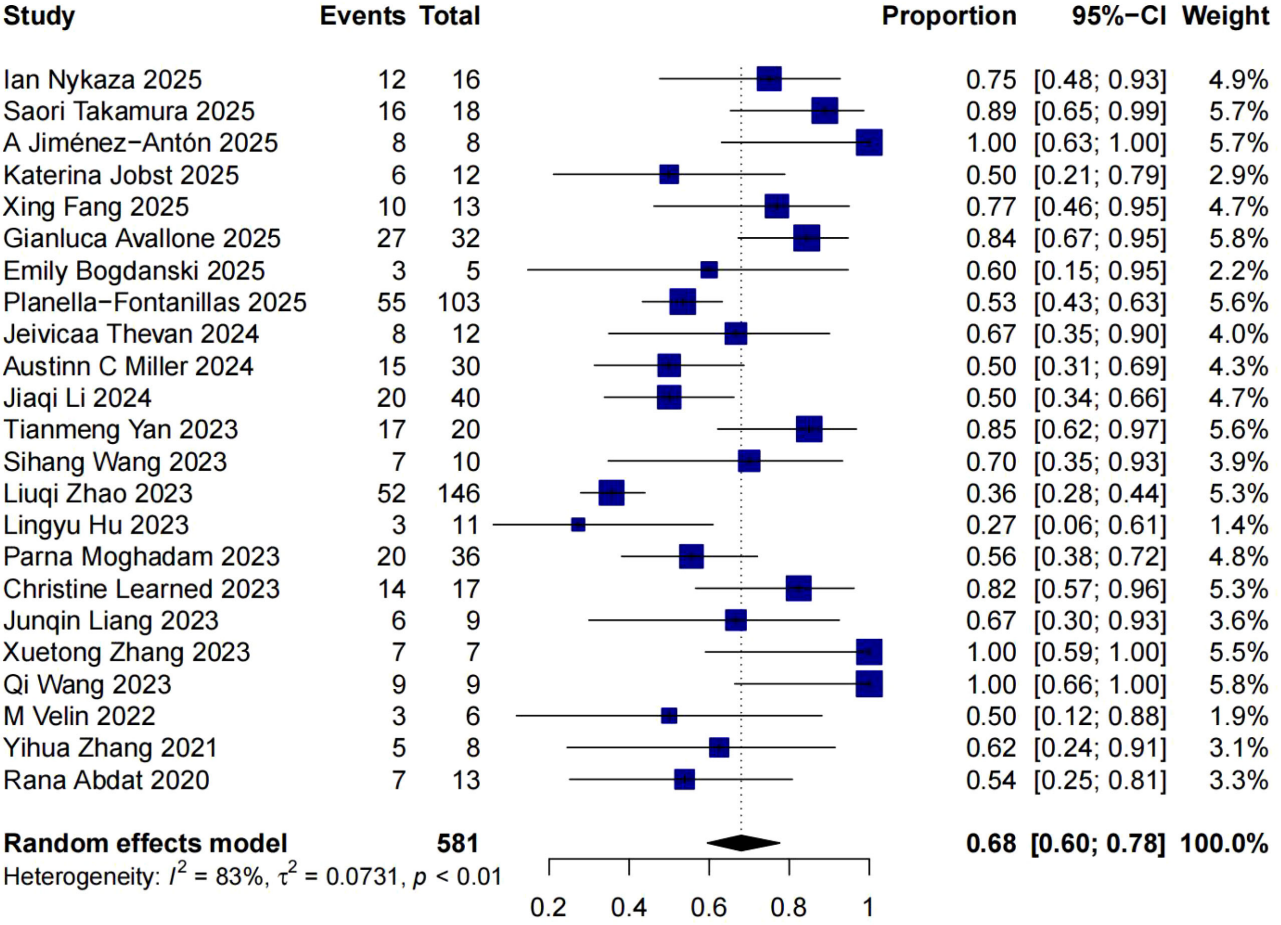
Pooled analysis of proportion of complete response with/without other systemic therapy.

The study of *Jinghui Li 2024* (n=6) did not report complete response rate (24). Therefore, a total of 23 studies on 581 patients were included for the pooled analysis of proportion of complete response with/without other systemic therapy. We observed a complete response rate of 68% (95% CI 60%∼78%) among these 581 patients (**Figure 2**).

**Figure 3:**
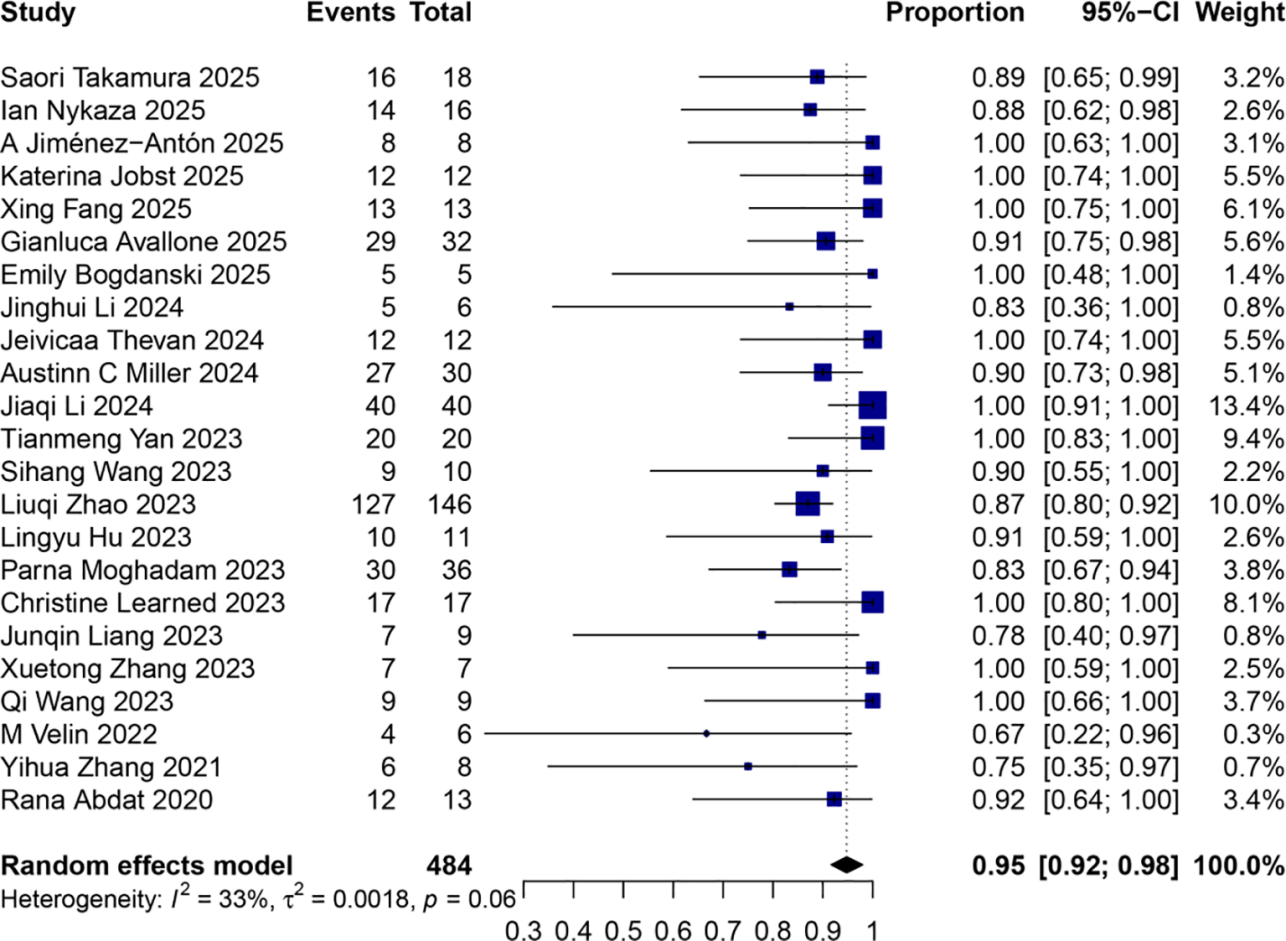
Pooled analysis of proportion of disease control with/without other systemic therapy.

The study of *Planella-Fontanillas 2024* (n=103) did not report disease control rate (25). Therefore, a total of 23 studies on 484 patients were included for the pooled analysis of proportion of disease control with/without other systemic therapy. We observed a disease control rate of 95% (95%CI 92%~98%) among these 484 patients (**Figure 3**).

**Figure 4:**
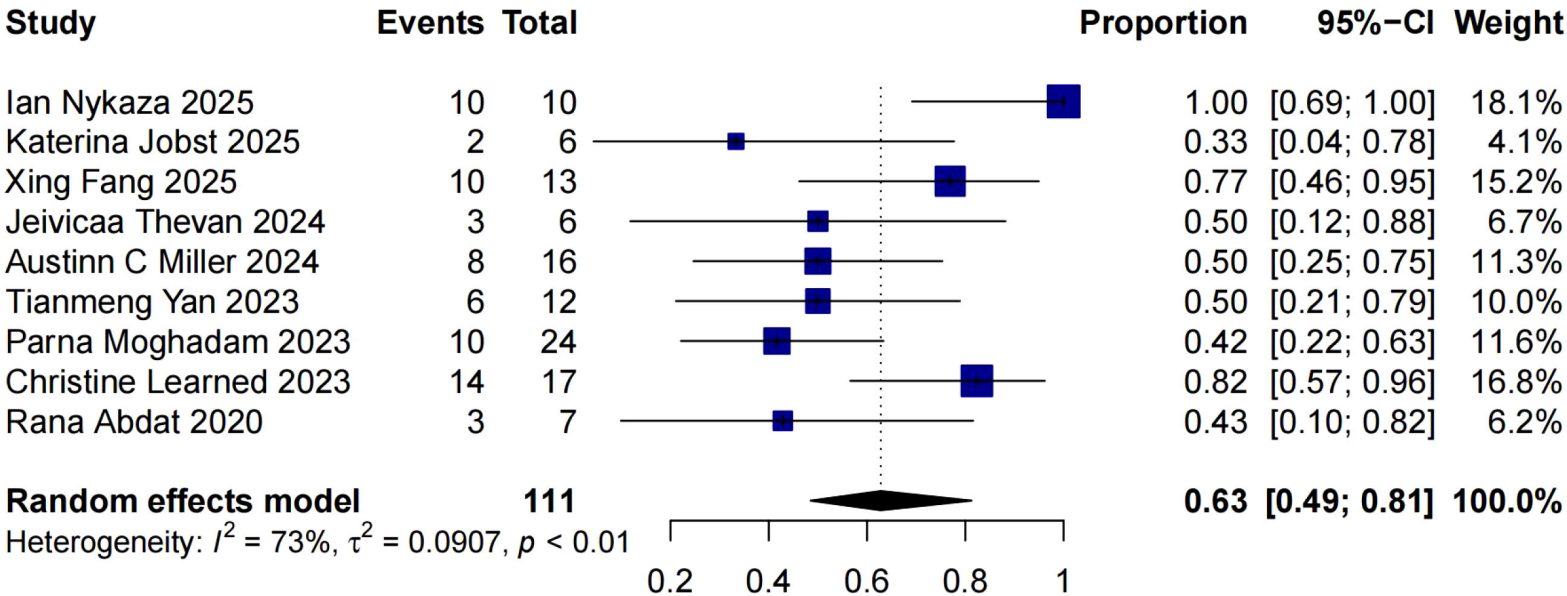
Pooled analysis of proportion of complete response without other systemic therapy.

Notably, 14 studies reported the treatment outcomes of patients with monotherapy of dupilumab. We excluded 5 studies in which number of participates using monotherapy less than 5 (24, 26–29). A total of 9 studies on 111 patients using dupilumab without other concomitant systemic therapy were included in the pooled analysis. We observed a complete response rate of 63% (95% CI 49%∼81%) among these 111 patients (**Figure 4**).

### 3.4 Safety

Two studies did not provide information on adverse events (*Jinghui Li 2024*, n=6; *Jiaqi Li 2024*, n=40). Safety data were available for 541 patients, and 82.1% (n=444/541) of them reported no adverse events. A total of 112 adverse events were reported in the remaining 97 patients. Most adverse events were mild and did not lead to treatment discontinuation. The most common adverse events were skin and soft-tissue infections (n=17), eosinophilia (n=11), and diabetes (n=10). However, diabetes was considered more likely to be related to concomitant use of systemic corticosteroids. During the follow-up period, 17 deaths were reported, and none of them were associated with dupilumab treatment. Reports of adverse events are summarized in **Table 2**.

**Table 2:** Adverse events.

Adverse event	N=112
Injection site reactions	4
Skin and soft-tissue infections	17
Psoriasis or psoriasis flare-up	6
Rosacea	1
Seborrhoeic dermatitis	1
Alopecia areata	1
Scabies	1
Asthenia	1
Drowsiness	1
Diarrhea	1
Fever of unknown origin	2
Hypokalemia	1
Eosinophilia	11
Thrombocytopenia	1
Conjunctivitis or keratitis	4
Intracerebral hemorrhage	1
Heart failure	1
Pericardial effusion	1
Pneumonia	9
Pleural effusion	1
Diabetes	10
Renal dysfunction	1
Deep vein thrombosis	2
Arthromyalgia	4
Osteoporosis	6
Thrombosis in lower extremities	1
Unknown cause of infection	5
Death	17
Cause of death	
COVID-19	2
Pneumonia	2
Pulmonary embolism	1
Cardiovascular event	2
Metastatic melanoma	2
Postvaccination sudden death	1
NR	7

NR, not reported.

## 4 Discussion

This systematic review examines the effectiveness and safety of dupilumab in BP treatment.

For the treatment of bullous pemphigoid, systemic corticosteroids represent a widely used therapeutic option, and this approach is recommended in several clinical practice guidelines (1, 5, 6). However, the long-term use of systemic corticosteroids is also associated with relapse and increased mortality. Off-label immunosuppressants (e.g. methotrexate, azathioprine, mycophenolate mofetil) and antibiotics with anti-inflammatory properties (e.g. doxycycline, minocycline) can also be used. However, all of them are associated with various side effects and limited therapeutic efficacy.

The mechanisms of BP development are not completely understood. However, mounting evidence indicates that a type 2 inflammatory response may play a key role in BP development. Studies demonstrate elevated levels of the type 2 cytokines IL-4, IL-5 and IL-13, elevated levels of the chemokine eotaxin-1 and greater numbers of eosinophils in BP lesions and peripheral blood as well as increased serum IgE in patients with BP (30). Dupilumab, a fully humanized monoclonal antibody, can block the shared receptor component for IL-4 and IL-13 which are key and central drivers for type 2 inflammation in multiple diseases.

Given these findings, dupilumab may be effective in BP. Several observational studies explored the use of dupilumab in BP. Cao et al. conducted a systemic review using descriptive statistics exploring efficiency of biologic treatment in BP in 2022 (31). In this systemic review, dupilumab led to complete remission in 66.7% (n=24/36) and partial remission in 19.4% (n=7/36) of patients without any reported adverse events in the 36 patients from 11 studies (including 7 case reports) included (31). What’s more, Abduelmula et al. also conducted a descriptive systemic review in 2022 (32). Abduelmula et al. included 39 patients from 12 studies (including 8 case reports) using dupilumab with/without other systemic therapy in BP, leading to complete remission in 82.1% (n=32/39) of patients (32). Previous reviews were systemic review using descriptive statistics which didn’t apply meta-analysis of treatment outcomes. Reporting only a single value of “overall effective rate” fails to reflect the impact of inter-study heterogeneity on the results. Our study applied weighted pooled analysis of “effective rate” from different studies and excluded studies with excessively small sample sizes (n<5)to control the quality of included studies. Meta-analysis can report standardized effect sizes and 95% confidence intervals (95% CI) so as to reflect the relative differences in treatment efficacy and the precision of the results.

Given the limited studies in this field, Nunes da Silva et al. performed a meta-analysis of comparative studies of dupilumab combined with corticosteroids and conventional corticosteroid therapy alone in patients with moderate-to-severe bullous pemphigoid (33). This meta-analysis included 127 patients from 4 non-randomized studies, demonstrating dupilumab combined with corticosteroids a greater reduction in BPDAI compared with patients who received conventional therapy (33).

Results from our study are consistent with previous reports. Our study included more recent studies from 2022 to 2025, and quantitatively synthesized data from these researches (including 23 retrospective studies and 1 case series). Pooled analysis revealed a complete response rate of 68% (95% CI 60%∼78%) and disease control rate of 95% (95% CI 92%~98%) in BP treated with dupilumab with/without other systemic therapy.

At present, dupilumab has no indication for bullous pemphigoid, and its clinical efficacy has not been verified in large-sample randomized controlled trials (RCTs). Therefore, it is often used in combination with oral glucocorticoids, immunosuppressants, or other therapies in clinical practice. However, in the studies included in our analysis, a subset of patients received dupilumab as the sole systemic therapeutic agent due to multiple factors such as elderly age or comorbidities, yet still achieved favorable clinical outcomes. Complete response rate achieved 63% (95% CI 49%∼81%) in patients with dupilumab without other systemic therapy.

Furthermore, we observed that 17.9% (n=97/541) of patients experienced adverse events (AEs), while most AEs were mild and did not lead to treatment discontinuation. Some of the AEs such as diabetes, osteoporosis and deep vein thrombosis are considered more related to concomitant use of systemic corticosteroids. Dupilumab generally have a strong safety profile for the treatment of BP.

Meanwhile, the first randomized controlled clinical trial (NCT04206553), designed to investigate the efficacy and safety of dupilumab in patients with BP is in progress (34). The results of this study will provide more information for the use of dupilumab in BP.

This study should be interpreted with several limitations. Information on specific demographic characteristics is not available for certain studies which is marked as “NR” in **Table 1**, **Supplementary Table S2**. If a study didn’t report data on a specific demographic characteristic, the data from that study shall be excluded from the descriptive statistics of this demographic characteristic. As a result, there may be a discrepancy between the statistical data and the actual baseline situation.

Moderate heterogeneity may affect the reliability of the results. The included studies were sourced from 7 distinct countries and encompassed multiple ethnic populations, thus introducing potential demographic heterogeneity. In addition, it should be noted that 408 patients (69.5%) among the included subjects were concurrently receiving other systemic therapies. Besides, efficacy assessments across the included studies were based on investigator-reported outcomes, and inter-investigator variability may have existed in the evaluation of cutaneous symptoms including erythema, vesiculation and pruritus. Finally, the included clinical studies varied in follow-up duration, which may also have resulted in differences in the observation of treatment efficacy and adverse events.

What’s more, publication bias is a common concern undermining the accuracy of meta-analyses. Studies with positive results are more likely to be published, while those with negative results may fail to be submitted or accepted. We plotted the funnel plot to assess the potential publication bias. As shown in **Supplementary files Figures S1-S3**, funnel plot asymmetry observed in this study suggests the potential non-publication of small-sample studies with negative findings. More large-sample, high-quality clinical studies should be performed in this field to further confirm the therapeutic efficacy of dupilumab.

No randomized controlled trials have yet been published in this field. Published studies are mostly single-center with limited sample size. And limited data on BP treated with dupilumab monotherapy is available. These limitations highlight the need for future studies to better address these issues.

## 5 Conclusion

This meta-analysis highlights the efficacy and safety of dupilumab in BP treatment, offering evidence for future clinical application. Future randomized controlled trials and well-designed observational studies with larger sample size are required to provide higher level evidence-based support.

## Data Availability

The original contributions presented in the study are included in the article/**Supplementary Material**. Further inquiries can be directed to the corresponding author.
