# Supplementary material for: Dupilumab treatment outcomes in bullous pemphigoid: a systematic review and single-arm meta-analysis
Source: Front Immunol. 2026 Feb 6;17:1651543. doi: 10.3389/fimmu.2026.1651543 (PMC12920225; doi:10.3389/fimmu.2026.1651543)

Supplementary Material

# Table S1. Search strategy

| **Pubmed（Oct. 20^th^, 2025)** | | |
| --- | --- | --- |
| 1 | Pemphigoid, Bullous[mesh] or Bullous Pemphigoid[tiab] or Pemphigoid[tiab] or Pemphigoids[tiab] | 9419 |
| 2 | dupilumab[mesh] or dupilumab[tiab] or Dupixent[tiab] | 4529 |
| 3 | Clinical Studies as Topic[Mesh] or Clinical Study[Publication Type] | 1573581 |
| 4 | Clinical Trials as Topic[Mesh] or Clinical Trial[Publication Type]) | 1369155 |
| 5 | Observational Studies as Topic[Mesh] or Observational Study[Publication Type] or case series[tiab] | 319248 |
| 6 | Retrospective Studies[Mesh] or Retrospective Study[tiab] | 1397777 |
| 7 | Randomized Controlled Trials as Topic[Mesh] or Randomized Controlled Trial[Publication Type] | 833077 |
| 8 | Cohort Studies[Mesh] or cohort study[tiab] | 2898151 |
| 9 | trial[mesh] or trial[tiab] or clinical stud[tiab] or stud[tiab] | 955056 |
| 10 | Case Reports as Topic[Mesh] or Case Reports[Publication Type] case report[tiab] or case series[tiab] | 2678366 |
| 11 | 3 or 4 or 5 or 6 or 7 or 8 or 9 or 10 | 6990139 |
| 12 | #1 and #2 and #11 | 86 |
| **Web of Science (Oct 20^th^, 2025)** | | |
| 1 | TS=(Pemphigoid, Bullous OR Bullous Pemphigoid OR bullous pemphigoid OR Pemphigoid OR pemphigoid OR pemphigoides) | 8767 |
| 2 | TS=(dupilumab OR Dupilumab OR Dupixent) | 6461 |
| 3 | TS=(clinical studies OR clinical study OR clinical trial* OR observational studies OR observational study OR case series OR retrospective studies OR retrospective study OR randomized controlled trial* OR cohort studies OR cohort study OR trial* OR clinical study OR clinical studies OR case report*) | 7017100 |
| 4 | #1 AND #2 AND #3 | 77 |
| **Embase (Oct. 20^th^, 2025)** | | |
| 1 | 'bullous pemphigoid'/exp OR 'pemphigoid, bullous':ab,ti OR 'bullous pemphigoid':ab,ti | 10416 |
| 2 | 'dupilumab'/exp OR dupilumab:ab,ti OR 'dupixent':ab,ti OR 'regn 668':ab,ti OR 'regn668':ab,ti OR 'sar 231893':ab,ti OR sar231893:ab,ti OR 'bat 2406':ab,ti OR 'bat2406':ab,ti | 11138 |
| 3 | 'clinical study'/exp OR 'clinical study':ab,ti OR 'clinical studies as topic':ab,ti OR 'clinical data':ab,ti OR 'medical trial':ab,ti | 14513554 |
| 4 | 'clinical trial'/exp OR 'clinical trial':ab,ti OR 'clinical drug trial':ab,ti OR 'major clinical trial':ab,ti OR 'trial, clinical':ab,ti | 2756072 |
| 5 | 'observational study'/exp OR 'observational study':ab,ti OR 'non experimental studies':ab,ti OR 'non experimental study':ab,ti OR 'nonexperimental studies':ab,ti OR 'nonexperimental study':ab,ti OR 'observation studies':ab,ti OR 'observation study':ab,ti OR 'observational studies':ab,ti OR 'observational studies as topic':ab,ti OR 'observational study as topic':ab,ti | 578717 |
| 6 | 'case study'/exp OR 'case study':ab,ti OR 'case series':ab,ti OR 'case studies':ab,ti OR 'large case series':ab,ti | 389708 |
| 7 | 'retrospective study'/exp OR 'retrospective study':ab,ti OR 'ex post facto design':ab,ti OR 'retrospective design':ab,ti OR 'retrospective panel studies':ab,ti OR 'retrospective panel study':ab,ti OR 'retrospective studies':ab,ti OR 'study, retrospective':ab,ti | 1978517 |
| 8 | 'randomized controlled trial'/exp OR 'controlled trial, randomized':ab,ti OR 'randomised controlled study':ab,ti OR 'randomised controlled trial':ab,ti OR 'randomized controlled study':ab,ti OR 'trial, randomized controlled':ab,ti OR 'randomized controlled trial':ab,ti | 1165682 |
| 9 | 'cohort analysis'/exp OR 'cohort analysis':ab,ti OR 'analysis, cohort':ab,ti OR 'cohort fertility':ab,ti OR 'cohort life cycle':ab,ti OR 'cohort studies':ab,ti OR 'cohort study':ab,ti OR 'fertility, cohort':ab,ti | 1574247 |
| 10 | #3 OR #4 OR #5 OR #6 OR #7 OR #8 OR #9 | 15073008 |
| 11 | #1 AND #2 AND #10 | 204 |
| **Cochrane (Oct. 20^th^ , 2025)** | | |
| 1 | MeSH descriptor: [Pemphigoid, Bullous] explode all trees | 68 |
| 2 | (Pemphigoid, Bullous):ti,ab,kw OR (bullous pemphigoid):ti,ab,kw OR (pemphigoid):ti,ab,kw OR (pemphigoids):ti,ab,kw | 218 |
| 3 | #1 OR #2 | 218 |
| 4 | dupilumab | 1464 |
| 5 | (dupilumab):ti,ab,kw OR (dupixent):ti,ab,kw | 1455 |
| 6 | #4 OR #5 | 1469 |
| 7 | MeSH descriptor: [Clinical Study] explode all trees | 42 |
| 8 | (clinical study):ti,ab,kw OR (Clinical Studies as Topic):ti,ab,kw | 925450 |
| 9 | #7 OR #8 | 925487 |
| 10 | MeSH descriptor: [Clinical Trial] explode all trees | 42 |
| 11 | (Clinical Trial):ti,ab,kw OR (Clinical Trials as Topic):ti,ab,kw OR (intervention study):ti,ab,kw | 1115111 |
| 12 | #10 OR #11 | 1115137 |
| 13 | MeSH descriptor: [Observational Study] explode all trees | 0 |
| 14 | (observational study):ti,ab,kw OR (Observational Studies as Topic):ti,ab,kw OR (case series):ti,ab,kw | 29362 |
| 15 | #13 OR #14 | 29362 |
| 16 | MeSH descriptor: [Retrospective Studies] explode all trees | 21092 |
| 17 | (Retrospective Studies):ti,ab,kw OR (Study, Retrospective):ti,ab,kw OR (Retrospective Study):ti,ab,kw OR (Studies, Retrospective):ti,ab,kw | 42472 |
| 18 | #16 OR #17 | 42472 |
| 19 | MeSH descriptor: [Randomized Controlled Trial] explode all trees | 34 |
| 20 | (Randomized Controlled Trial):ti,ab,kw OR (Randomized Controlled Trials as Topic):ti,ab,kw | 819437 |
| 21 | #19 OR #20 | 819437 |
| 22 | MeSH descriptor: [Cohort Studies] explode all trees | 210861 |
| 23 | (cohort studies):ti,ab,kw OR (cohort study):ti,ab,kw | 77107 |
| 24 | #22 OR #23 | 264538 |
| 25 | #9 OR #12 OR #15 OR #18 OR #21 OR #24 | 1459529 |
| 26 | #3 AND #6 AND #25 | 7 |

# Table S2. Characteristics of included studies

| **Study** | **Region** | **Study design** | **No. of patients using dupilumab** | **Patients using dupilumab without other systemic therapy** | **Female, n** | **Age, y** | **Disease duration, m** |
| --- | --- | --- | --- | --- | --- | --- | --- |
| **Gianluca Avallone 2025** | Italy (multicenter) | Retrospective study | 32 | 10 | 19 | 76.0±14.3 | 21.0±21.7 |
| **Emily Bogdanski 2025** | U.S. | Case series | 5 | 0 | 1 | 74.0±6.4 | NR |
| **Ian Nykaza 2025** | U.S. | Retrospective study | 16 | 10 | 7 | 72.7 ±11.2 | 1.9 (range: 0.6～27.9) |
| **Saori Takamura 2025** | Japan | Retrospective study | 18 | 0 | 9 | 78 | NR |
| **A Jiménez-Antón 2025** | Spain | Retrospective study | 8 | 0 | 4 | 78.5 (range: 65.75～85.5) | 5.5 (range: 2～11) |
| **Katerina Jobst 2025** | Germany | Retrospective study | 12 | 6 | 5 | 78.6 (range: 67～93) | NR |
| **Xing Fang 2025** | China | Retrospective study | 13 | 13 | 4 | 89.85±5.64 | 2.5 (range: 0.5～120) |
| **Jeivicaa Thevan 2024** | U.S. | Retrospective study | 12 | 6 | 4 | 76.0±15.0 | 31.5±19.2 |
| **Nidia Planella-Fontanillas 2025** | Spain (multicenter) | Retrospective study | 103 | 22 | 43 | 77.3 (IQR 66.4～84.2) | 59.3 (IQR 33.8～111.3). |
| **Austinn C Miller 2024** | U.S. | Retrospective study | 30 | 16 | 15 | 75.3±10.0 | 3.5 (IQR 1.0～42.0) |
| **Jinghui Li 2024** | China | Retrospective study | 6 | 1 | 1 | 69.7±10.5 | NR |
| **Jiaqi Li 2024** | China | Retrospective study | 40 | 0 | 19 | 74.25±10.0 | 2.6 (IQR 1～3) |
| **Tianmeng Yan 2023** | China (multicenter) | Retrospective study | 20 | 12 | 9 | 73.4±10.65 | 10.25±16.91 |
| **Lingyu Hu 2023** | China | Retrospective study | 11 | 1 | 7 | 75.9±12.8 | 3.5 (IQR 1.8～7.5) |
| **Parna Moghadam 2023** | France (multicenter) | Retrospective study | 36 | 24 | NR | NR | NR |
| **Christine Learned 2023** | U.S.  (multicenter) | Retrospective study | 17 | 17 | 7 | 72.2±15.2 | 4.0 (IQR 2.5～17.5) |
| **Liuqi Zhao 2023** | China (multicenter) | Retrospective study | 146 | 31 | 60 | 73 (IQR 64～85) | 3.9 (IQR 1.7～12.9) |
| **Sihang Wang 2023** | China | Retrospective study | 10 | 1 | 3 | 72.7±17.5 | 4.5 (IQR 2.0～9.8) |
| **Junqin Liang 2023** | China | Retrospective study | 9 | 0 | 2 | 69.3±15.9 | 6.0 (IQR 1.8～42.5) |
| **Qi Wang 2023** | China | Retrospective study | 9 | 0 | 4 | 72.0 (IQR 71.0～81.5) | 4.5 (IQR 0.84～10.0） |
| **Xuetong Zhang 2023** | China | Retrospective study | 7 | 1 | 4 | 73.4±10.2 | 6.0 (IQR 2.0～24.0) |
| **M Velin 2022** | France | Retrospective study | 6 | 1 | 4 | 78.6±6.0 | 5.5 (IQR 2.1～25.0) |
| **Yihua Zhang 2021** | China | Retrospective study | 8 | 0 | 5 | 64.5(IQR 45.5～71.75) | 2 (IQR 1.25～49.5) |
| **Rana Abdat 2020** | U.S.  (multicenter) | Retrospective study | 13 | 7 | 5 | 76.8±10.3 | 18.0 (IQR 3.0～45.0) |

Abbreviation: BP, bullous pemphigoid; NR, not reported.

# Table S3. Risk of Bias Assessment.

| **Study** | **Types of bias** | | | | | | | **Overall rating** |
| --- | --- | --- | --- | --- | --- | --- | --- | --- |
|  | **Bias due to confounding** | **Bias in selection of participants into the study** | **Bias in classification of interventions** | **Bias due to deviations from intended interventions** | **Bias due to missing data** | **Bias due to measurement of outcomes** | **Bias in selection of the reported result** |  |
| **Gianluca Avallone 2025** | Moderate | Low | Moderate | Low | Low | Moderate | Low | Moderate |
| **Emily Bogdanski 2025** | Moderate | Low | Moderate | Low | Low | Moderate | Low | Moderate |
| **Ian Nykaza 2025** | Moderate | Low | Moderate | Low | Low | Moderate | Low | Moderate |
| **Saori Takamura 2025** | Moderate | Low | Moderate | Low | Moderate | Moderate | Low | Moderate |
| **Katerina Jobst 2025** | Moderate | Low | Moderate | Low | Low | Moderate | Low | Moderate |
| **Xing Fang 2025** | Moderate | Low | Moderate | Low | Low | Moderate | Low | Moderate |
| **A Jiménez-Antón 2025** | Moderate | Low | Moderate | Low | Moderate | Moderate | Low | Moderate |
| **Jeivicaa Thevan 2024** | Moderate | Low | Moderate | Low | Low | Moderate | Low | Moderate |
| **Nidia Planella-Fontanillas 2025** | Moderate | Low | Moderate | Low | Moderate | Moderate | Low | Moderate |
| **Austinn C Miller 2024** | Moderate | Low | Moderate | Low | Low | Moderate | Low | Moderate |
| **Jinghui Li 2024** | Moderate | Low | Moderate | Low | Moderate | Moderate | Low | Moderate |
| **Jiaqi Li 2024** | Moderate | Low | Moderate | Low | Moderate | Moderate | Low | Moderate |
| **Tianmeng Yan 2023** | Moderate | Low | Moderate | Low | Low | Moderate | Low | Moderate |
| **Lingyu Hu 2023** | Moderate | Low | Moderate | Low | Low | Moderate | Low | Moderate |
| **Parna Moghadam 2023** | Moderate | Low | Moderate | Low | Low | Moderate | Low | Moderate |
| **Christine Learned 2023** | Moderate | Low | Moderate | Low | Low | Moderate | Low | Moderate |
| **Liuqi Zhao 2023** | Moderate | Low | Moderate | Low | Low | Moderate | Low | Moderate |
| **Sihang Wang 2023** | Moderate | Low | Moderate | Low | Low | Moderate | Low | Moderate |
| **Junqin Liang 2023** | Moderate | Low | Moderate | Low | Low | Moderate | Low | Moderate |
| **Xuetong Zhang 2023** | Moderate | Low | Moderate | Low | Low | Moderate | Low | Moderate |
| **Qi Wang 2023** | Moderate | Low | Moderate | Low | Low | Moderate | Low | Moderate |
| **M Velin 2022** | Moderate | Low | Moderate | Low | Low | Moderate | Low | Moderate |
| **Yihua Zhang 2021** | Moderate | Low | Moderate | Low | Low | Moderate | Low | Moderate |
| **Rana Abdat 2020** | Moderate | Low | Moderate | Low | Low | Moderate | Low | Moderate |

**4 Figure S1. Funnel plot for pooled analysis of proportion of complete response with/without other systemic therapy.**


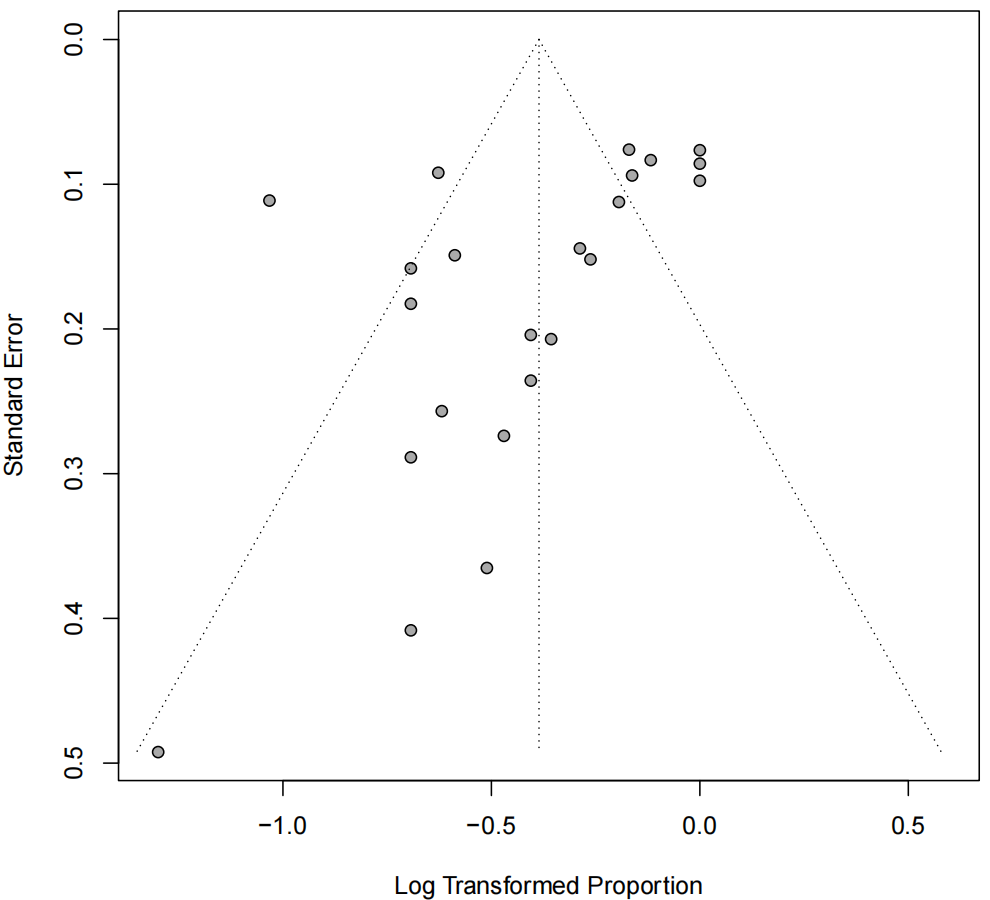


**5 Figure S2. Funnel plot for pooled analysis of proportion of disease control with/without other systemic therapy.**


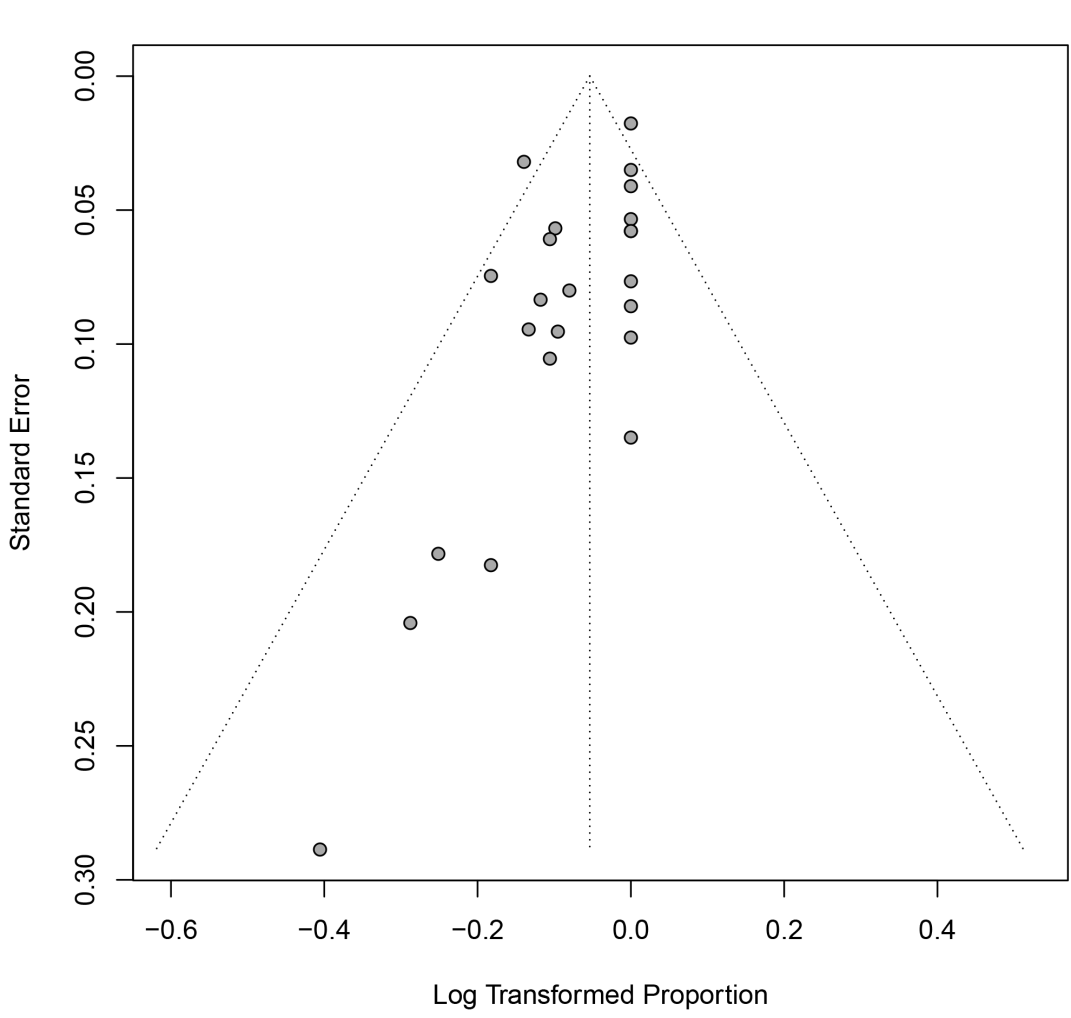


**6 Figure S3. Funnel plot for pooled analysis of proportion of complete response without other systemic therapy.**


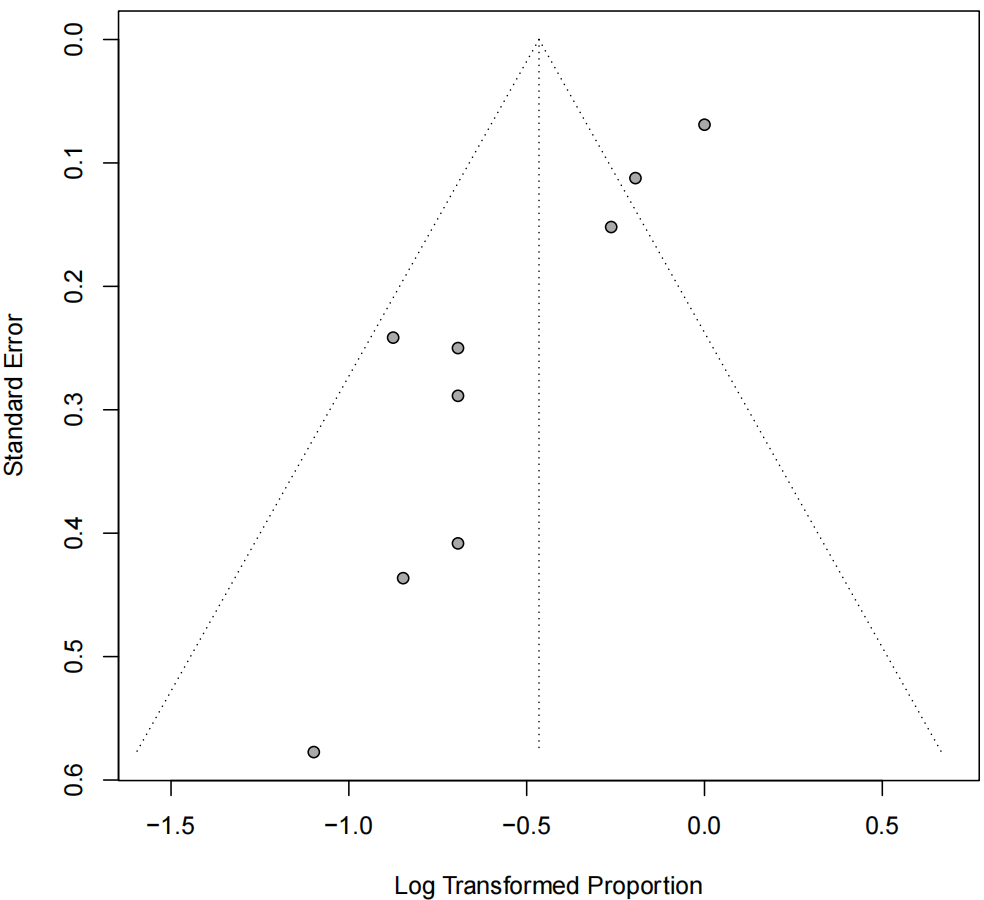

Supplement: Supplementary file 1 [file DataSheet1.docx]
